# The Influence of Trehalose on Atherosclerosis and Hepatic Steatosis in Apolipoprotein E Knockout Mice

**DOI:** 10.3390/ijms20071552

**Published:** 2019-03-28

**Authors:** Aneta Stachowicz, Anna Wiśniewska, Katarzyna Kuś, Anna Kiepura, Anna Gębska, Mariusz Gajda, Magdalena Białas, Justyna Totoń-Żurańska, Kamila Stachyra, Maciej Suski, Jacek Jawień, Ryszard Korbut, Rafał Olszanecki

**Affiliations:** 1 Chair of Pharmacology, Jagiellonian University Medical College, 31-531 Krakow, Poland; anna.niepsuj@uj.edu.pl (A.W.); katarzyna.1.kus@uj.edu.pl (K.K.); a.kiepura@uj.edu.pl (A.K.); mfgebska@cyf-kr.edu.pl (A.G.); justyna.toton-zuranska@uj.edu.pl (J.T.-Ż.); k.stachyra@doctoral.uj.edu.pl (K.S.); maciej.suski@uj.edu.pl (M.S.); jacek.jawien@uj.edu.pl (J.J.); r.korbut@uj.edu.pl (R.K.); rafal.olszanecki@uj.edu.pl (R.O.); 2 Department of Histology, Jagiellonian University Medical College, 33-332 Krakow, Poland; mmgajda@cyf-kr.edu.pl; 3 Chair of Pathomorphology, Jagiellonian University Medical College, 31-531 Krakow, Poland; magdalena.bialas@uj.edu.pl

**Keywords:** atherosclerosis, fatty liver, trehalose, autophagy, apoE-knockout mice

## Abstract

Atherosclerosis and nonalcoholic fatty liver disease (NAFLD) are frequent causes of death in the Western countries. Recently, it has been shown that autophagy dysfunction plays an important role in the pathogenesis of both atherosclerosis and NAFLD; thus, activators of autophagy might be useful for novel therapeutic interventions. Trehalose—a naturally occuring disaccharide present in plants, bacteria, fungi, insects, and certain types of shrimps—is a known inducer of autophagy. However, according to the literature, its anti-atherosclerotic and anti-steatotic potential seem to depend on the experimental setting. The aim of our study was to comprehensively describe the influence of a prolonged treatment with orally administered trehalose on the development of atherosclerotic lesions and hepatic steatosis in apolipoprotein E knockout (apoE^−/−^) mice in an experimental set up reflecting both moderate and severe proatherogenic conditions: male apoE^−/−^ mice on a chow diet (CD) and female apoE^−/−^ mice fed with a high-fat diet (HFD). We found that exogenous trehalose inhibited atherosclerosis and attenuated hepatic steatosis in apoE^−/−^ mice. Such effects of trehalose were not associated with changes of plasma cholesterol, low-density lipoproteins (LDL), or high-density lipoproteins (HDL). Moreover, the anti-steatotic action of trehalose in the liver was associated with the induction of autophagy. The exact molecular mechanisms of both the anti-atherosclerotic action of trehalose and its inhibitory effect on liver steatosis require further clarification.

## 1. Introduction

Atherosclerosis and nonalcoholic fatty liver disease (NAFLD) are currently a worldwide public health problem and among the frequent causes of death in the Western countries [1]. Atherosclerosis is a complex inflammatory disease of the arterial wall, frequently associated with dyslipidemia, characterized by endothelial activation, an accumulation of oxidized low-density lipoproteins, subsequent migration of monocytes and other inflammatory cells to the subendothelial space, and their excessive apoptosis/necrosis. Such processes progressively lead to the formation of atherosclerotic lesions with lipid-rich necrotic cores and rupture-prone fibrous caps [2,3]. A growing body of evidence indicates that NAFLD is a key independent risk factor for atherosclerosis development [4]. Worldwide, this complex common liver disorder is manifested by triglyceride accumulation in the cytoplasm of hepatocytes and encompasses a wide spectrum of pathological conditions ranging from simple hepatic steatosis to steatosis with inflammatory response, nonalcoholic steatohepatitis (NASH), cirrhosis and fibrosis, and finally hepatocarcinoma [5]. 

Recently, it has been shown that autophagy dysfunction plays an important role in the pathogenesis of both atherosclerosis and NAFLD [6,7]. Autophagy is an evolutionarily conserved and self-protecting catabolic process of degradation of damaged organelles and misfolded proteins. It uses many molecular pathways (in general, mammalian target of rapamycin (mTOR)-dependent and/or mTOR-independent—for review, see reference [8]), contributes to the inhibition of apoptosis and inflammation and the promotion of cholesterol efflux and efferocytosis (non-inflammatory cell death and removal) in atherosclerotic lesions, as well as to the reduction of triglyceride levels in hepatocytes [9,10]. Thus, activators of autophagy might be useful for therapeutic interventions in atherosclerosis and NAFLD. Trehalose—a non-reducing, naturally occurring disaccharide, which consists of two glucose units linked in an α,α-1,1-glycosidic linkage—is one of the known inducers of autophagy [11]. It is widely distributed in the biological world, being common in plants, bacteria, fungi, insects, and certain types of shrimps, where it serves as a protectant against environmental stresses, such as cold, heat, oxidation, and dehydration [12,13]. Interestingly, trehalose is also a popular stabilizer, sweetener, and humectant used in confectionery, beverages, bakery goods, rice, and frozen food, as it has a lower sweetening intensity than sucrose and decreases the freezing point of food [14]. The dietary intakes of trehalose are 5.7–9.7 g/day, as estimated for the populations of the USA and Australia, and could reach 19 g occasionally (according to WHO Food Additives Series 46: Trehalose). Noteworthy, despite the presence of trehalose-degrading enzyme (trehalase) in the mammalian small intestine, orally administered trehalose has been proved to exert significant biological effects in mouse models of obesity, fructose-induced lipid steatosis, muscular dystrophy, and Huntington’s disease [15,16,17,18]). Surprisingly, so far, in animal models of atherosclerosis (high-fat diet-fed apolipoprotein E knockout mice and high-cholesterol-fed rabbits), the action of trehalose was observed only when administered parenterally [19,20]. Thus, the anti-atherosclerotic action of trehalose seems to be critically dependent on the severity of the model and the experimental setting. 

The apoE^−/−^ mice are a widely utilized animal model of atherosclerosis, since they spontaneously develop arterial lesions, dyslipidemia, and hypercholesterolemia, as well as changes in the liver resembling mild hepatic steatosis [21,22]. The apoE^−/−^ mice are considered a versatile model to study the variability of the anti-atherosclerotic action of a given substance, as the development of atherosclerotic plaques and hepatic steatosis in these animals varies depending on age, sex, and diet and is the least severe in male mice on a chow diet and the most severe in female mice on a high-fat diet [23,24,25]. 

Thus, the aim of our study was to comprehensively describe the influence of a 16-week treatment with orally administered trehalose on the progression of atherosclerotic lesions and hepatic steatosis in apoE^−/−^ mice in an experimental set-up reflecting both moderate and severe proatherogenic conditions: in male apoE^−/−^ mice on a chow diet (CD) and female apoE^−/−^ mice fed with a high-fat diet (HFD) to determine whether oral administration of this disaccharide and under what conditions might affect atherosclerosis and hepatic steatosis. 

## 2. Results

### 2.1. The Influence of Trehalose on Atherosclerosis

**Figure 1:**
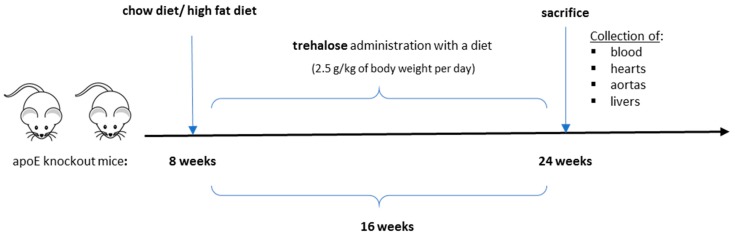
The experimental design of the study. Blue arrows indicate the beginning and end of the experiment; black arrow indicates the timeline.

**Figure 2:**
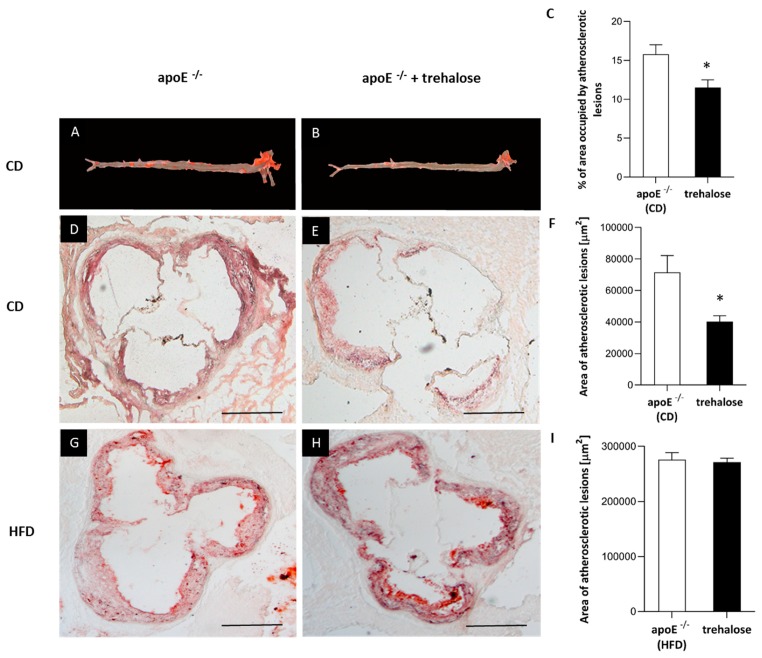
Atherosclerotic lesions in trehalose-treated apoE^−/−^ mice. Sudan IV-stained representative aortas in apoE^−/−^ mice on a CD (**A**) and trehalose-treated apoE^−/−^ mice on a CD (**B**). Representative micrographs showing oil-red O-stained atherosclerotic lesions measured by the atherosclerosis evaluation at the aortic sinus in the aorta of apoE^−/−^ mice on a CD (**D**), trehalose-treated apoE^−/−^ mice on a CD (**E**), apoE^−/−^ mice on an HFD (**G**), and trehalose-treated apoE^−/−^ mice on an HFD (**H**). Quantitative analysis of atherosclerotic lesions areas in apoE^−/−^ mice and trehalose-treated apoE^−/−^ mice on a CD or an HFD measured by the en face (**C**) or cross section methods (**F,I**) (mean ± SEM; * *p* < 0.05 as compared to apoE^−/−^ mice on a CD; *n* = 7–11 per group). Scale bars = 500 µm.

**Figure 3:**
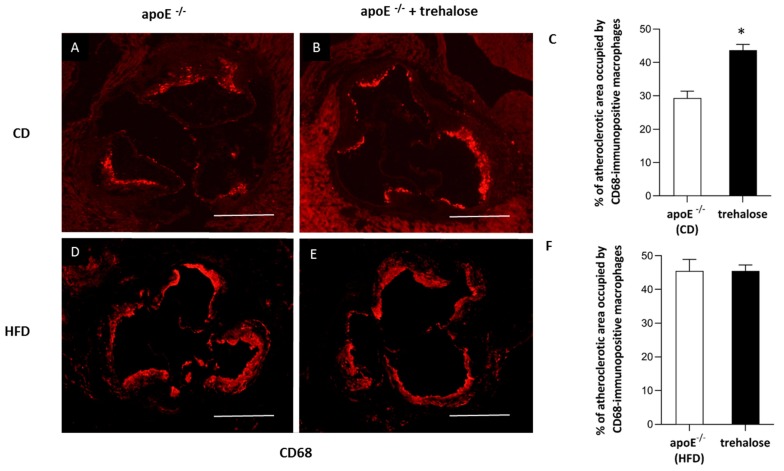
Macrophage infiltrated in the atherosclerotic lesion of trehalose-treated apoE^−/−^ mice. Immunohistochemical staining of aortic roots showing CD68-positive macrophages (**A,B,D,E**) in apoE^−/−^ mice on a CD (**A**), trehalose-treated apoE^−/−^ mice on a CD (**B**), apoE^−/−^ mice on an HFD (**D**), and trehalose-treated apoE^−/−^ mice on an HFD (**E**). Quantitative analysis of atherosclerotic lesions areas occupied by CD68-positive macrophages in apoE^−/−^ mice and trehalose-treated apoE^−/−^ mice on a CD and an HFD (**C,F**) (mean ± SEM; * *p* < 0.05 as compared to apoE^−/−^ mice on a CD; *n* = 7–11 per group). Scale bars = 500 µm.

**Figure 4:**
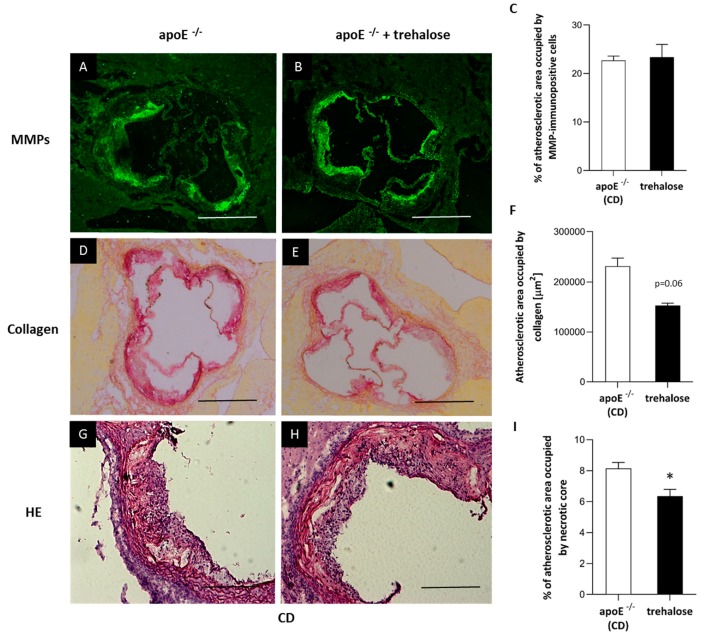
Content of metalloproteinases, collagen and necrotic core in the atherosclerotic lesion of trehalose-treated apoE^−/−^ mice. Immunohistochemical staining showing non-specific activity of gelatinases (mostly MMP-2 and MMP-9) (**A,B**), total collagen (**D,E**) and necrotic core (**G,H**) in atherosclerotic lesions of apoE^−/−^ mice on a CD and trehalose-treated apoE^−/−^ mice on a CD. Quantitative analysis of atherosclerotic lesions areas occupied by non-specific activity of gelatinases (mostly MMP-2 and MMP-9), total collagen, and necrotic core in apoE^−/−^ mice and trehalose-treated apoE^−/−^ mice on a CD (**C,F,I**) (mean ± SEM; * *p* < 0.05 as compared to apoE^−/−^ mice on a CD; *n* = 7–11 per group). Scale bars = 500 µm.

**Figure 5:**
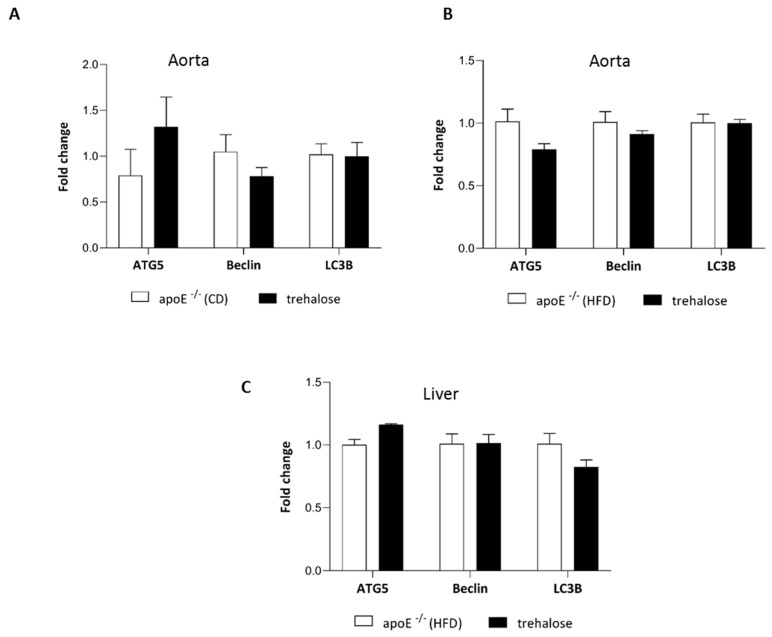
Influence of trehalose on autophagy in the aorta and liver of apoE^−/−^ mice. Expression level of genes related to autophagy: *ATG5*, *Beclin*-1, and *LC3B* in the aorta of apoE^−/−^ mice and trehalose-treated apoE^−/−^ mice on a CD (**A**) and on an HFD (**B**) and in the liver of apoE^−/−^ mice and trehalose-treated apoE^−/−^ mice on na HFD (**C**) (mean ± SEM; n = 4–5 per group).

The experimental design of our study is presented in Figure 1. There were no trehalose treatment-related signs of toxicity in apoE^−/−^ mice. As compared to untreated animals, treatment with trehalose did not change the daily water or food intake in apoE^−/−^ mice and did not influence the body weight of animals on a CD (32.2 ± 1.7 g vs. 32.1 ± 2.8 g) and an HFD (26.6 ± 1.2 g vs. 25.7 ± 1.8 g). Trehalose caused a significant decrease of atherosclerotic lesions by almost 40% in the aorta of apoE^−/−^ mice fed a CD as measured by the en face method (11.5 ± 0.9% vs 15.8 ± 1.2%; *p* < 0.05) (Figure 2A,B,C) and atherosclerosis evaluation at the aortic sinus (40118 ± 3904 μm^2^ vs 71492 ± 10703 μm^2^; *p* < 0.05) (Figure 2D,E,F). Moreover, trehalose treatment significantly increased the macrophage content as evidenced by CD68 staining (43.7% ± 1.7% vs 29.3% ± 2%; *p* < 0.05) (Figure 3A,B,C) and decreased the necrotic core as measured by the hematoxylin–eosin (HE) method (6.4% ± 0.4% vs 8.1% ± 0.4%; *p* < 0.05) (Figure 4G,H,I) in atherosclerotic plaques of apoE^−/−^ mice on a CD. However, it did not change the non-specific activity of gelatinases (mostly matrix metalloproteinases: MMP-2 and MMP-9) and the total collagen content as measured by in situ zymography (23.4% ± 2.66% vs 22.7% ± 0,9%; *p* > 0.05) and picro-sirius red staining (152993 ± 4490 μm^2^ vs 230862± 16329 µm^2^; *p* = 0.059), respectively (Figure 4D,E,F). On the contrary, trehalose influenced neither the progression of atherosclerosis (“cross section”: 271325.4 ± 7225.5 μm^2^ vs 275830.8 ± 12676.9 μm^2^; *p* > 0.05) (Figure 2G,H,I) nor the content of macrophages (CD68 staining: 45.25% ± 1.7% vs 45.52% ± 3.4%; *p* < 0.05) (Figure 3D,E,F) in apoE^−/−^ mice fed an HFD. Real-time PCR experiments did not reveal any significant changes of expression of genes related to autophagy (*LC3B*, *ATG5*, *Beclin*) in the aorta of apoE^−/−^ mice on CD or HFD upon trehalose administration (Figure 5A,B).

### 2.2. The Influence of Trehalose on Hepatic Steatosis

**Figure 6:**
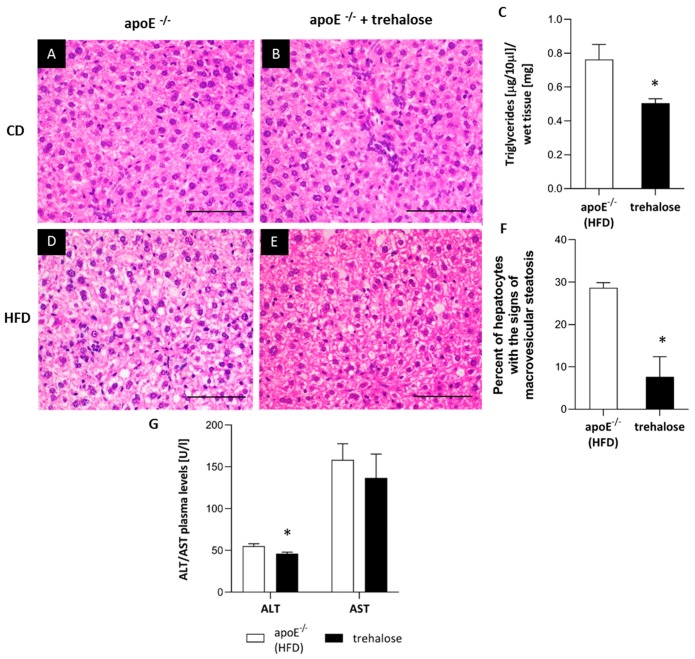
Influence of trehalose on hepatic steatosis in apoE^−/−^ mice. Representative images of the livers of apoE^−/−^ mice on a CD (**A**), trehalose-treated apoE^−/−^ mice on a CD (**B**), apoE^−/−^ mice on an HFD (**D**), and trehalose-treated apoE^−/−^ mice on an HFD (**E**). The images show hematoxylin and eosin staining (**A,B,D,E**) and quantitative analysis of macrovesicular steatosis (**C**), as well as TG content in the liver (**F**) and plasma alanine aminotransferase (ALT)/ aspartate aminotransferase (AST) levels in apoE^−/−^ mice and trehalose-treated apoE^−/−^ mice on an HFD (**G**). Magnification 40x (mean ± SEM; * *p* < 0.05 as compared to apoE^−/−^ mice on a HFD; *n* = 3 or *n* = 7 per group). Scale bars = 50 µm.

The HE staining did not reveal major disturbances of the liver structure in both untreated and trehalose-treated apoE^−/−^ mice on a CD. Portal spaces were not enlarged and did not present inflammatory infiltrates; only some hepatocytes had signs of granulation in the cytoplasm (Figure 6A,B). On the contrary, the cytoplasm of hepatocytes in the liver of apoE^−/−^ mice on n HFD had a granular structure with signs of macrovesicular steatosis in about 30% of hepatocytes present in all three lobular zones. The lobular structure of the liver was still preserved, and the portal spaces were minimally enlarged and devoid of inflammatory infiltrates (Figure 6C). The treatment with trehalose caused a reduction of macrovesicular steatosis, evident in about 7% of hepatocytes, mostly in the first zone (Figure 6D,E). Moreover, trehalose administration resulted in a significant decrease of triglycerides level by about 35% in the liver of apoE^−/−^ mice on an HFD (Figure 6F). In addition, trehalose treatment significantly decreased plasma alanine aminotransferase (ALT) level in apoE^−/−^ mice on an HFD (Figure 6G). Trehalose did not change the plasma levels of total cholesterol, low-density lipoproteins (LDL), and high-density lipoproteins (HDL) in apoE^−/−^ mice on a CD and an HFD (Table 1). Importantly, the treatment with trehalose resulted in a significant decrease of plasma triglycerides (TG) level only in apoE^−/−^ mice on an HFD (Table 1). 

### 2.3. The Influence of Trehalose on Autophagy in The Liver

**Figure 7:**
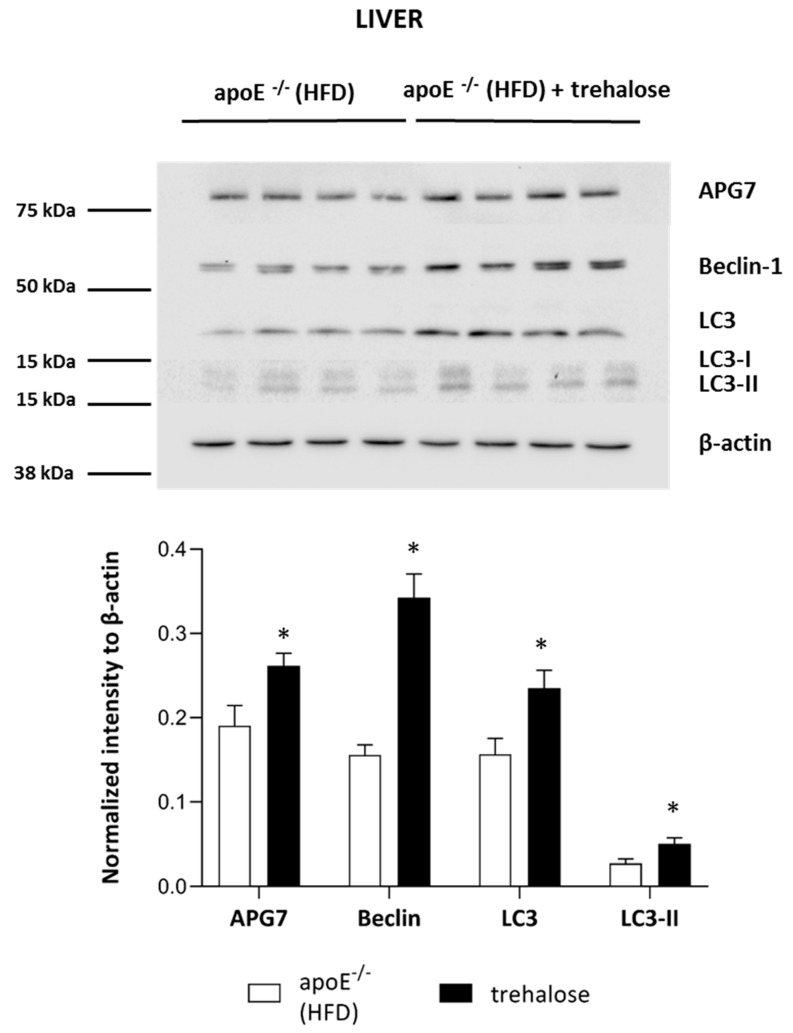
Influence of trehalose on autophagy in the liver of apoE^−/−^ mice. Expression level of APG7, Beclin-1, LC3, LC3-II, and β-actin in the liver of apoE^−/−^ mice and trehalose-treated apoE^−/−^ mice on an HFD (mean ± SEM; * *p* < 0.05 as compared to apoE^−/−^ mice on an HFD; *n* = 4 per group).

To investigate the molecular mechanisms responsible for the reduction of hepatic steatosis upon trehalose treatment in the liver of apoE^−/−^ mice on an HFD, we performed immunoblotting and real-time PCR of key factors related to autophagy: Beclin-1, microtubule-associated proteins 1A/1B light-chain 3B (LC3), and autophagy-related protein 7 (APG7). As compared to the untreated mice, the administration of trehalose resulted in a significant increase in the protein expression of Beclin-1, LC3, LC3-II, and APG7 in the liver of apoE^−/−^ mice on an HFD (Figure 7). However, real-time PCR experiments did not reveal any significant change of expression of genes related to autophagy (*LC3B*, *ATG5*, *Beclin*) in the liver of apoE^−/−^ mice on an HFD upon trehalose administration (Figure 5C).

## 3. Discussion

Several lines of evidence indicate that the impairment of autophagy contributes to the pathogenesis of atherosclerosis and fatty liver disease and the pharmacological modulation of autophagy could protect against the progression of these disorders [6,7]. In the present work, we have shown that a known autophagy inducer—trehalose—given orally for a 16-week period and without any visible adverse effects, inhibited atherosclerosis and attenuated hepatic steatosis in apoE^−/−^ mice fed with a chow and a high-fat diet, respectively. Noteworthy, such actions of trehalose were not associated with changes of plasma cholesterol, LDL, or HDL. 

### 3.1. The Influence of Trehalose on Atherosclerosis

In our hands, in the experimental layout corresponding to a gentle model of atherosclerosis, i.e., in a CD-fed male apoE^−/−^ mice, trehalose caused a 40% reduction of atherosclerotic plaques. Interestingly, this was accompanied by an increased content of macrophages and decreased necrotic cores in atherosclerotic lesions of apoE^−/−^ mice. Recently, it has been shown that a macrophage-specific dysfunction of autophagy is a hallmark of atherosclerotic lesions and contributes to atherogenesis by the increased accumulation of protein aggregates, defective efferocytosis, and impaired lipid degradation. Moreover, it has been shown that the stimulation of the macrophage autophagy-lysosomal system through pharmacological or genetic interventions could be atheroprotective [9,19,26]. Although the increased content of macrophages in atherosclerotic lesions may seem contradictory to the anti-atherosclerotic action of trehalose, it could, in theory, reflect autophagy-related inhibition of macrophage apoptosis and/or necrosis and enhanced macrophage autophagy-lysosomal biogenesis, as the same effect was observed in atherosclerotic plaques upon treatment with rapamycin, an mTOR pathway-dependent activator of autophagy [27]. Which macrophage phenotype (M1/M2) predominates and whether, in terms of inflammatory reaction, trehalose may increase the number of truly “silent” macrophages in the plaques require, however, further studies. In contrast to apoE^−/−^ mice on a chow diet, administration of trehalose to female apoE^−/−^ mice fed with a high-fat diet did not influence either the progression of atherosclerosis or the content of macrophages in the atherosclerotic plaques. Interestingly, Sergin et al. showed that combined trehalose administration (i.p., 2 g/kg three times per week and orally, 3% in drinking water) reduced the atherosclerotic lesions in apoE^−/−^ mice fed an HFD [19]. However, they concluded that the intraperitoneal administration of trehalose was responsible for this effect, as the oral treatment alone did not decrease the atherosclerotic lesions in apoE^−/−^ mice on an HFD. Thus, our results are in keeping with their data. The question arises why the anti-atherosclerotic effect of oral trehalose was attenuated on an HFD. One could speculate about the reduction of trehalose absorption from the gastrointestinal (GI) tract caused by the HFD itself or changes in the main GI trehalose metabolic pathways affecting trehalase levels or the bacterial flora [8]. However, the significant effect of trehalose treatment on liver steatosis in animals fed with an HFD denies this hypothesis. Taking into account the presence of the enzyme trehalase in the intestinal mucosa, which rapidly hydrolyses trehalose, the concentration of trehalose within the aortic wall could be too low to inhibit atherogenesis in such an aggressive model as female apoE^−/−^ mice on an HFD.

### 3.2. The Influence of Trehalose on Hepatic Steatosis

The liver structure of apoE^−/−^ mice on a CD did not reveal significant disturbances; some hepatocytes had signs of granulation of the cytoplasm. The treatment with trehalose did not change the appearance of the liver of apoE^−/−^ mice on a CD. On the contrary, the cytoplasm of hepatocytes in the liver of apoE^−/−^ mice on an HFD had a granular structure with signs of macrovesicular steatosis in about 30% of cells. Importantly, the treatment with trehalose caused a reduction of steatosis, which was evident in about 7% of hepatocytes. Such an effect was accompanied by a significant decrease of triglyceride levels in the liver and plasma. Moreover, trehalose seemed to exert a hepatoprotective effect, as it significantly diminished plasma ALT level in apoE^−/−^ mice on an HFD. 

**Figure 8:**
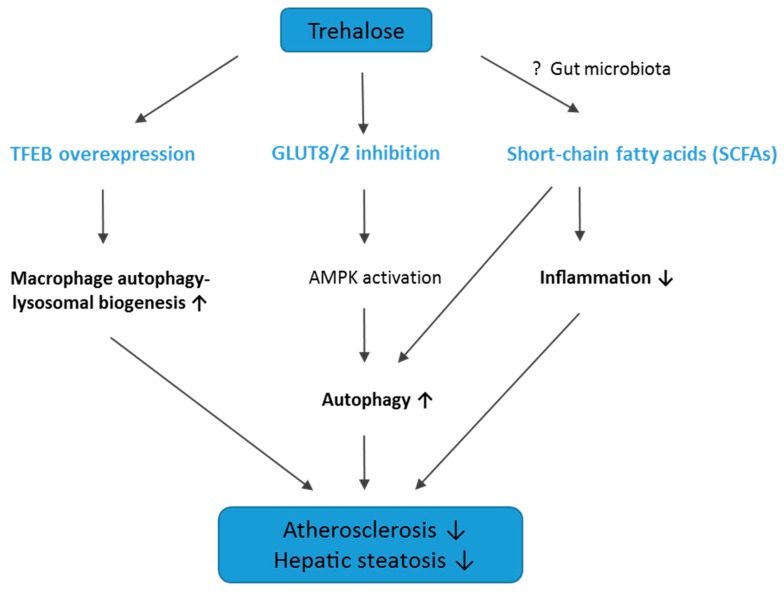
Graphical summary of the potential mechanisms of action of trehalose in atherosclerosis and hepatic steatosis. TFEB, transcription factor EB; GLUT8/2, glucose transporter 8/2; AMPK, AMP-activated protein kinase; SCFAs, short-chain fatty acids. ↑, upregulation; ↓, downregulation; ?, potentially involved pathway.

The mechanism of trehalose action in the attenuation of hepatic steatosis in apoE^−/−^ mice on an HFD seems to be at least partially related to autophagy induction, as evidenced by the significant increase in key proteins related to autophagy in the liver: LC3, Beclin-1, and APG7. In keeping with our results, DeBosch et al. showed that trehalose administration reduced high-fructose diet-induced hepatic steatosis in wild-type C57BL/6J mice through the inhibition of glucose transport via the activation of AMP-activated protein kinase (AMPK)-dependent autophagy [15,28]. In addition, it was observed that trehalose supplementation can have also other advantageous effects in metabolic diseases: it mitigated insulin resistance, suppressed mesenteric adipocyte hypertrophy, increased the plasma level of adiponectin in obese mice, and reduced hepatic endoplasmic reticulum stress in old mice [16,29,30,31]. Thus, it is tempting to speculate that this disaccharide provides potentially a novel therapeutic approach to the treatment/prevention of fatty liver diseases and metabolic syndrome. However, the exact mechanisms of the beneficial actions of trehalose require further studies. The potential mechanisms of action of trehalose in atherosclerosis and hepatic steatosis are depicted in Figure 8.

### 3.3. Conclusions, Limitations of The Study, and Future Directions

We have found that trehalose, given orally for a 16-week period, was able to inhibit atherosclerosis only in the less aggressive model examined (CD-fed male apoE^−/−^ mice). Such effects of trehalose were not associated with changes of plasma cholesterol, LDL, or HDL. Interestingly, while inefficient in terms of inhibition of atherosclerosis, oral trehalose was able to attenuate hepatic steatosis even in an aggressive model of the disease (HFD-fed apoE^−/−^ mice). The anti-steatotic action of trehalose in the liver was associated with the induction of autophagy. Yet, the exact molecular mechanisms of both the anti-atherosclerotic action of trehalose and its inhibitory effect on liver steatosis require further clarification. 

So far, oral administration of trehalose has proved inefficient, which was referred to the presence of the enzyme trehalase in the intestinal mucosa, kidney, liver, and blood of almost all mammals. Trehalase rapidly hydrolyses trehalose to glucose, which could lead to a decrease of trehalose bioavailability [32,33]. On the other hand, many studies have shown that orally administered trehalose was present in the serum up to 4 h after intake and might exert therapeutic effects [15,16,17,18]. Moreover, some reports have indicated that trehalose is incompletely digested by trehalase in the small intestine [34]. Likely, unabsorbed trehalose could be transformed to short-chain fatty acids (SCFAs) by the intestinal microflora. Interestingly, it has been shown that SCFAs, such as acetate, propionate, and butyrate, induce autophagy [35] and have anti-inflammatory properties, which could be protective in atherosclerosis [36]. However, we did not measure the level of trehalose and SCFAs in the blood; therefore, whether the mechanism of action of trehalose is related to SCFAs requires further investigations. 

Taking into account all the above limitations, future pharmacological interventions based on the anti-atherosclerotic action of this disaccharide should consider the combined use of trehalose with trehalase inhibitors or degradation-resistant trehalose analogs. 

## 4. Materials and Methods 

### 4.1. Animal Experiments

All animal procedures were performed conforming the guidelines from Directive 2010/63/EU of the European Parliament on the protection of animals used for scientific purposes and approved by the Jagiellonian University Ethical Committee on Animal Experiments (no. 73/2011). Eighteen male apoE–knockout mice and 18 female apoE–knockout (apoE^−/−^) mice on the C57BL/6J background were purchased from Taconic (Ejby, Denmark). The animals were kept on 12 h dark/12 h light cycles in air-conditioned rooms (22.5 ± 0.5 °C, 50 ± 5% humidity) with access to water ad libitum and diet. The mice were put on a chow (CD) or a high-fat (HFD) diet made by Morawski (Kcynia, Poland) at the age of 8 weeks for 16 weeks. The percentage composition of CD and HFD, showing differences in the content of fat and cholesterol, is presented in Table 2. Four groups of animals were studied: male apoE^−/−^ mice on chow diet (*n* = 10), male apoE^−/−^ mice on chow diet treated with trehalose (*n* = 8), female apoE^−/−^ mice on high-fat diet (*n* = 11), female apoE^−/−^ mice on high-fat diet treated with trehalose (*n* = 7). Trehalose (α-D-Glucopyranosyl-α-D-glucopyranoside, Sigma-Aldrich, Saint-Louis, MO, USA) was mixed without heating with the same diet and administered to mice at a dose of 2.5 g per kg of body weight per day. The dose of trehalose (2.5 g/kg of body weight per day) was chosen according to the Food and Drug Administration (FDA) rules of animal-to-human equivalents and was relevant to the average daily intake of trehalose estimated for the human population [37]. The animals were injected with 1000 IU of fraxiparine i.p (Sanofi-Synthelabo, Paris, France) at the age of 6 months and killed in a chamber filled with carbon dioxide. Next, the blood was collected, and aortas, hearts, and livers were dissected.

### 4.2. Atherosclerosis Studies

Atherosclerosis development in apoE^−/−^ mice was assessed using the en face method and atherosclerosis evaluation at the aortic sinus, as described previously [38]. Briefly, 10 micrometer-thick serial cryosections were stained with Meyer’s hematoxylin and oil red-O, picro-sirius red, and HE (Sigma-Aldrich, Saint-Louis, MO, USA) and analyzed under an Olympus BX50 (Olympus, Tokyo, Japan) microscope with the LSM Image Browser software (Zeiss, Jena, Germany). 

### 4.3. Immunohistochemistry of Aortic Roots

For indirect immunohistochemistry, acetone-fixed sections of the ascending aorta were used. The sections were preincubated overnight with 5% non-immunogenic goat serum with 2% fat-free milk to block nonspecific binding of antibodies. Incubation with the primary antibody rat anti-mouse CD68 (Serotec, Kidlington, UK) (dilution 1:800) was performed overnight at room temperature in wet chambers. Then, goat anti-rat IgG biotinylated antibody followed by DTAF-conjugated streptavidin (Jackson IR, Cambridgeshire, UK) was applied to visualize the rat antibody. Sections were examined using an epifluorescence Olympus BX50 microscope (Olympus, Tokyo, Japan) equipped with appropriate filter cubes DTAF fluorescence. The images were registered with a Camedia DP71 digital camera (Olympus, Tokyo, Japan). In each section, the total area occupied by CD68-immunopositive macrophages was measured using the LSM Image Browser software (Zeiss, Jena, Germany). Non-specific activity of gelatinases (mostly MMP-2 and MMP-9) in tissue sections was demonstrated by in situ zymography. Unfixed sections were thawed and incubated for 2 h at 37 °C in a dark humid chamber with a reaction buffer containing 50 mg/mL of FITC-labelled DQ-gelatin (Invitrogen, Carlsbad, CA, USA). The sections were rinsed in PBS and fixed in 4% formaldehyde for 5 min, then mounted in glycerin/PBS. The fluorescence of gelatinase activity was observed under a BX50 microscope (Olympus, Tokyo, Japan) equipped with a mercuric burner and an appropriate filter set (U-MNIBA) and recorded with a DP71 camera (Olympus, Tokyo, Japan). The area displaying fluorescence resulting from the enzymatic break-down of fluorescein isothiocyanate (FITC)-gelatin represented local tissue gelatinase activity. Areas of MMP activity as well as intensities of fluorescence (reflecting enzyme activity; pixel intensity/green channel: range 0–255) were measured in eight sections from each sample, applying the LSM Image Browser software [38].

### 4.4. Histology of The Liver

The liver tissue samples were formalin fixed and embedded in paraffin, and 2 µm-thick paraffin sections were stained with the HE method as described previously [21].

### 4.5. Biochemical Methods

Plasma was centrifuged at 1000× *g* at 4 °C for 10 min and stored at −80 °C. The levels of TG, LDL, HDL, and total cholesterol were measured using commercial kits (Roche Molecular Biochemical, Pleasanton, CA, USA). Moreover, the plasma levels of aspartate aminotransferase (AST) and alanine aminotransferase (ALT) were assayed by the Reflovet Plus equipment (Roche, Basel, Switzerland) using commercial kits: Reflotron GOT, Reflotron GPT (Roche, Basel, Switzerland). TG levels in the liver were assayed using the Triglyceride Colorimetric Assay Kit (Cayman Chemical, Ann Arbor, MI, USA), according to the manufacturer’s instructions.

### 4.6. Western Blot

Immunoblotting experiments were performed as previously described [39]. The following primary antibodies were used: 1:1000 ANTI-Beclin 1 (Abcam, Cambridge, UK), 1:10000 ANTI-APG7 (Santa Cruz, Dallas, TX, USA), 1:500 ANTI-LC3A/B (Abcam, Cambridge, UK), 1:5000 ANTI-β-actin (Sigma, Saint-Louis, MO, USA). Images were recorded by the ImageQuant Las 500 (GE Healthcare, Chicago, IL, USA) and analyzed using Image Lite Studio software (LI-COR, Lincoln, NE, USA).

### 4.7. Real-Time PCR

Real-time PCR experiments to determine the expression levels of genes related to autophagy (*ATG5*, *Beclin*, *LC3B*) in the aorta of untreated and trehalose-treated apoE^−/−^ mice on a CD as well as an HFD were conducted as previously described [39]. Relative gene expression analysis with *ACTB* (Bio Rad, Hercules, CA, USA) as reference gene was performed using the 7900HT fast real-rime PCR System (Applied Biosystems, Foster City, CA, USA). The sequences of the designed primers were as follows: 5′-CAC TGC TCT GTC TTG TGT AGG TTG-3′ (forward) and 5′-TCG TTG TGC CTT TAT TAG TGC ATC-3′ (reverse) for LC3B; 5′-ACC ACA AGC AGC TCT GGA TGG GAC T-3′ (forward) and 5′-GCC GCT CCG TCG TGG TCT GAT AT-3′ (reverse) for ATG5; 5′-GGG GTT TGC GGT TTT TCT GGG AC-3′ (forward) and 5′-TCT CCA CGT CCA TCC ATC CTG TAC GGA AG -3′ (reverse) for Beclin. The data were analyzed using the 2^–∆∆Ct^ method by Data Assist v3.01 software (Applied Biosystems, Foster City, CA, USA).

### 4.8. Statistics

Results are presented as a mean + SEM. The equality of variance and the normality of the data were checked and then the nonparametric Mann –Whitney U test or t-test were used for statistical analysis of the data (Statistica 10, StatSoft, Krakow, Poland); *p* < 0.05 was considered as statistically significant.

## Figures and Tables

**Table 1:** Plasma levels of total cholesterol, high-density lipoproteins (HDL), low-density lipoproteins (LDL), and triglycerides (TG), presented as mean ± SD; * *p* < 0.05 as compared to the apoE^−/−^ (HFD) group; *n* = 4 per group.

Group	Total Cholesterol [mmol/L]	HDL [mmol/L]	LDL [mmol/L]	TG [mmol/L]
**apoE^−/−^ (CD)**	13.4 ± 0.6	3.72 ± 0.95	8.24 ± 0.84	1.36 ± 0.22
**apoE^−/−^ (CD) + trehalose**	12.0 ± 3.20	3.58 ± 0.30	7.4 ± 0.88	1.48 ± 0.57
**apoE^−/−^ (HFD)**	27.24 ± 4.49	3.93 ± 0.83	22.51 ± 4.10	1.03 ± 0.22
**apoE^−/−^ (HFD) + trehalose**	25.25 ± 4.77	5.06 ± 0.50	19.90 ± 4.49	0.67 ± 0.12 *

**Table 2:** Percentage composition of a chow diet (CD) and a high-fat (HFD) diet.

Components	Chow Diet (CD)	High Fat Diet (HFD)
**Fat**	4%	15.2%
**Cholesterol**	0%	0.25%
**Protein**	17%	16.9%
**Fiber**	6.5%	5.4%
**Ash**	5.6%	5.3%
**Others**	66.9%	57%

## Abbreviations

CD	chow diet
HE	hematoxylin/eosin
HFD	high-fat diet
NAFLD	nonalcoholic fatty liver disease
